# Supplementary figures and images for: Negative Modulation of Macroautophagy by Stabilized HERPUD1 is Counteracted by an Increased ER-Lysosomal Network With Impact in Drug-Induced Stress Cell Survival
Source: Front Cell Dev Biol. 2022 Mar 2;10:743287. doi: 10.3389/fcell.2022.743287 (PMC8924303; doi:10.3389/fcell.2022.743287)

**HeLa Herpud1- $\Delta$ ULD**

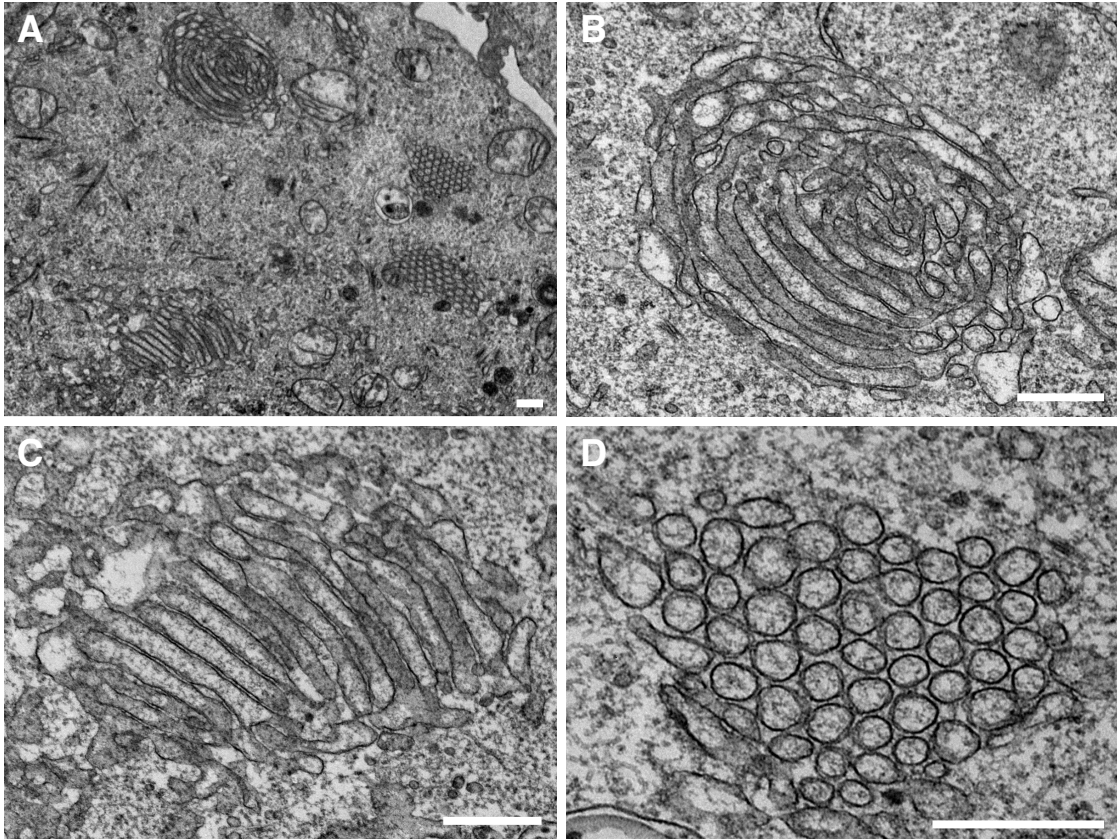

**Bar: 500 nm**

**HeLa Herpud1- $\Delta$ ULD**

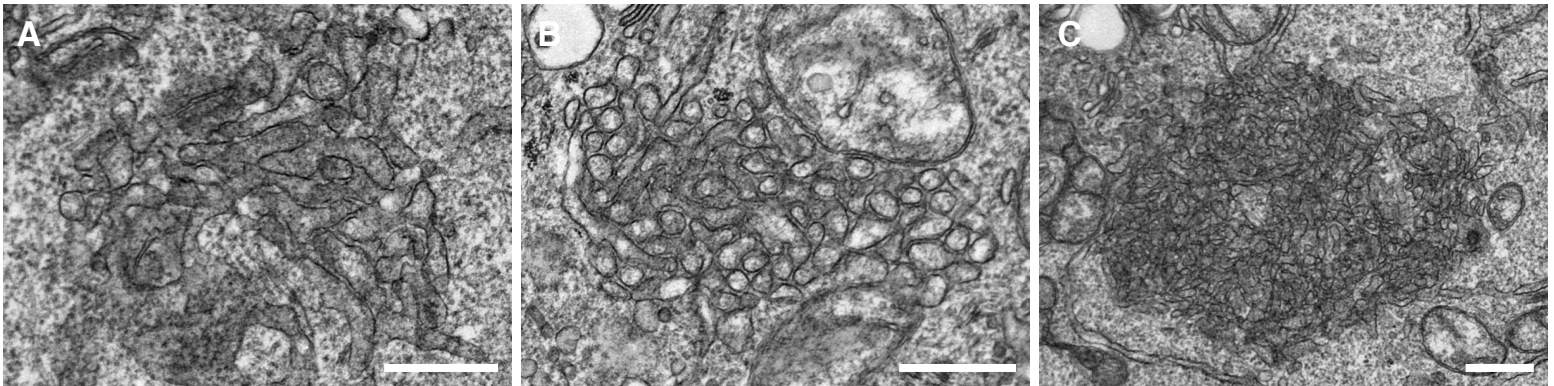

**Bar: 500 nm**

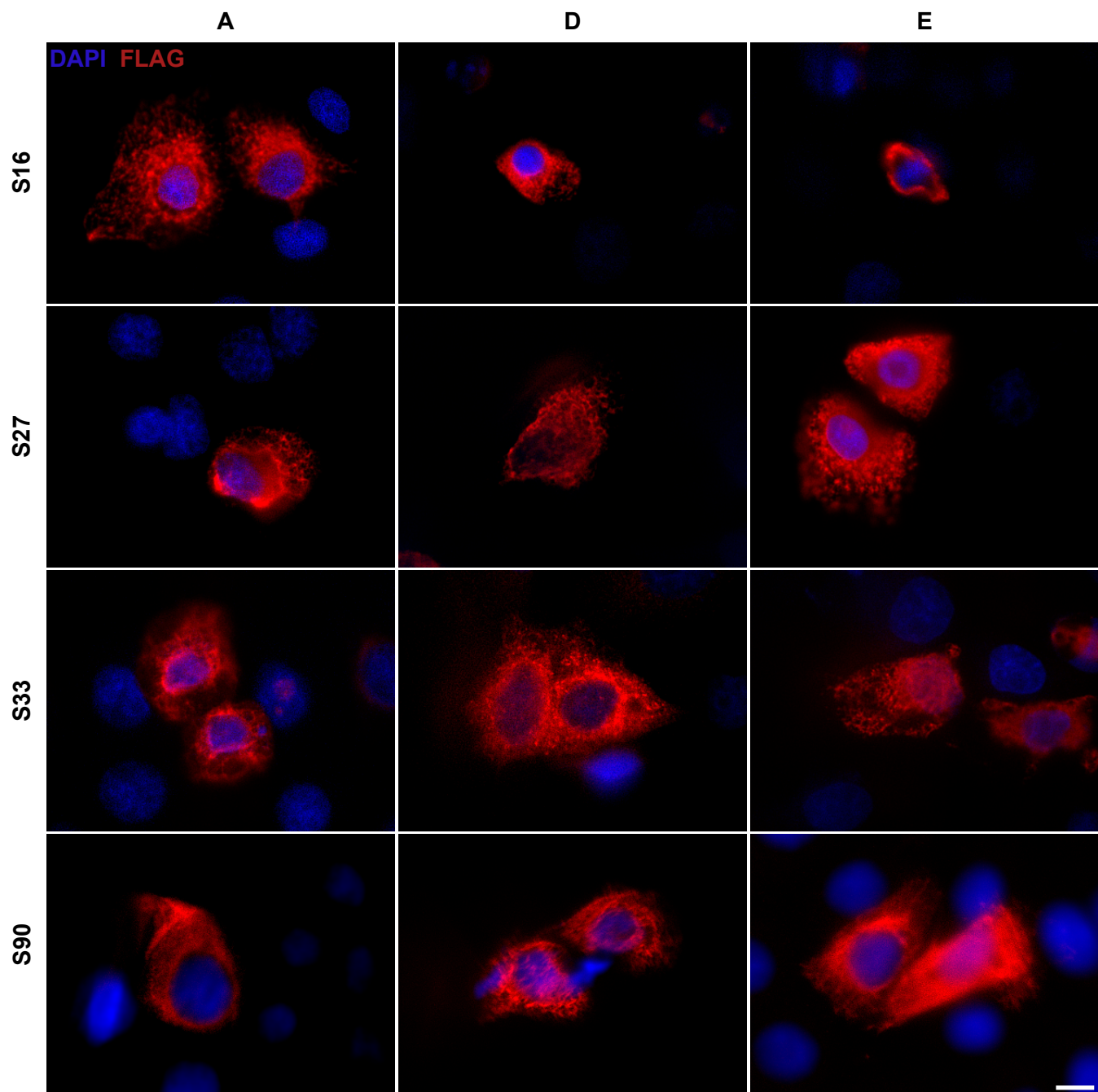

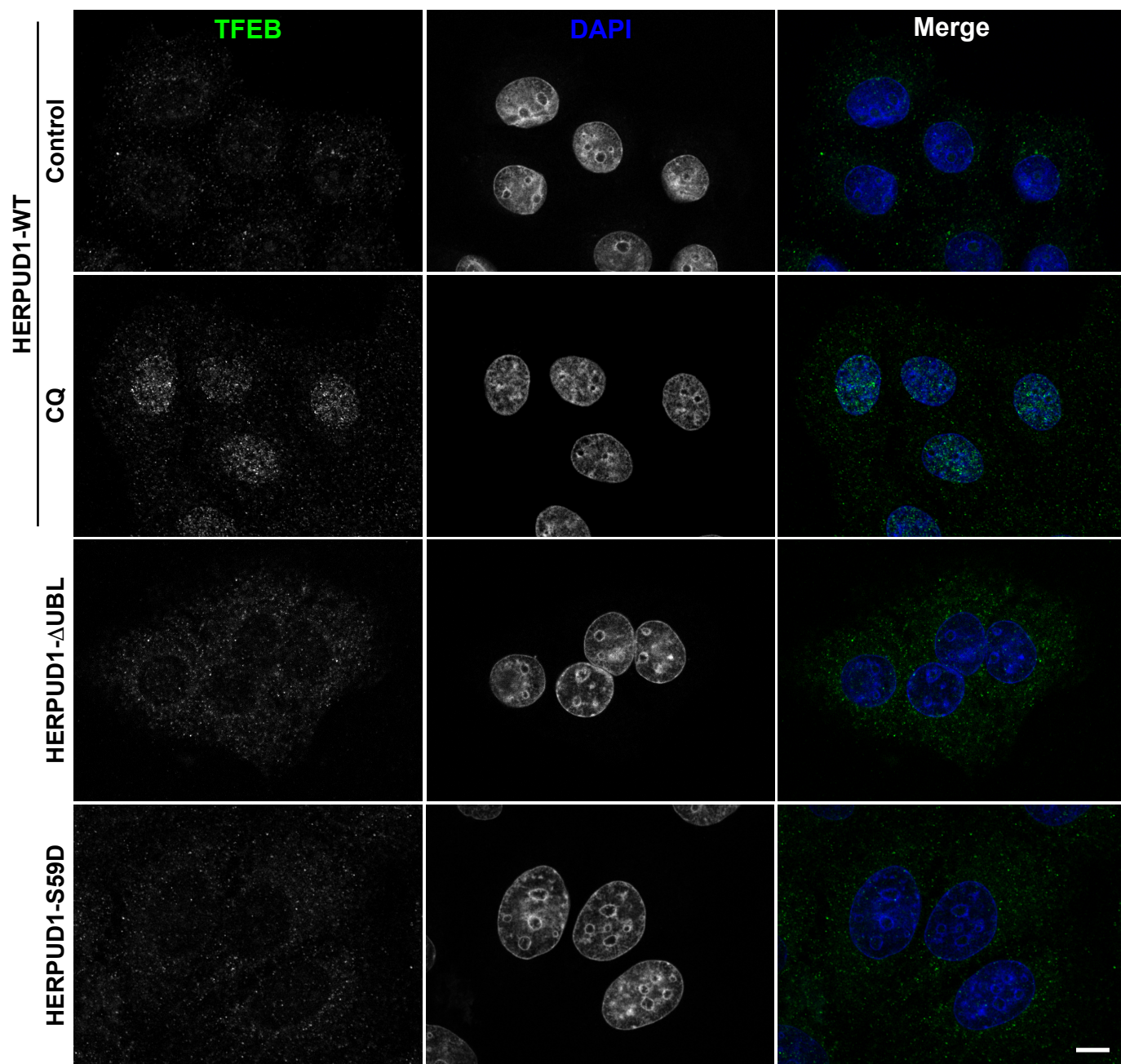

**B**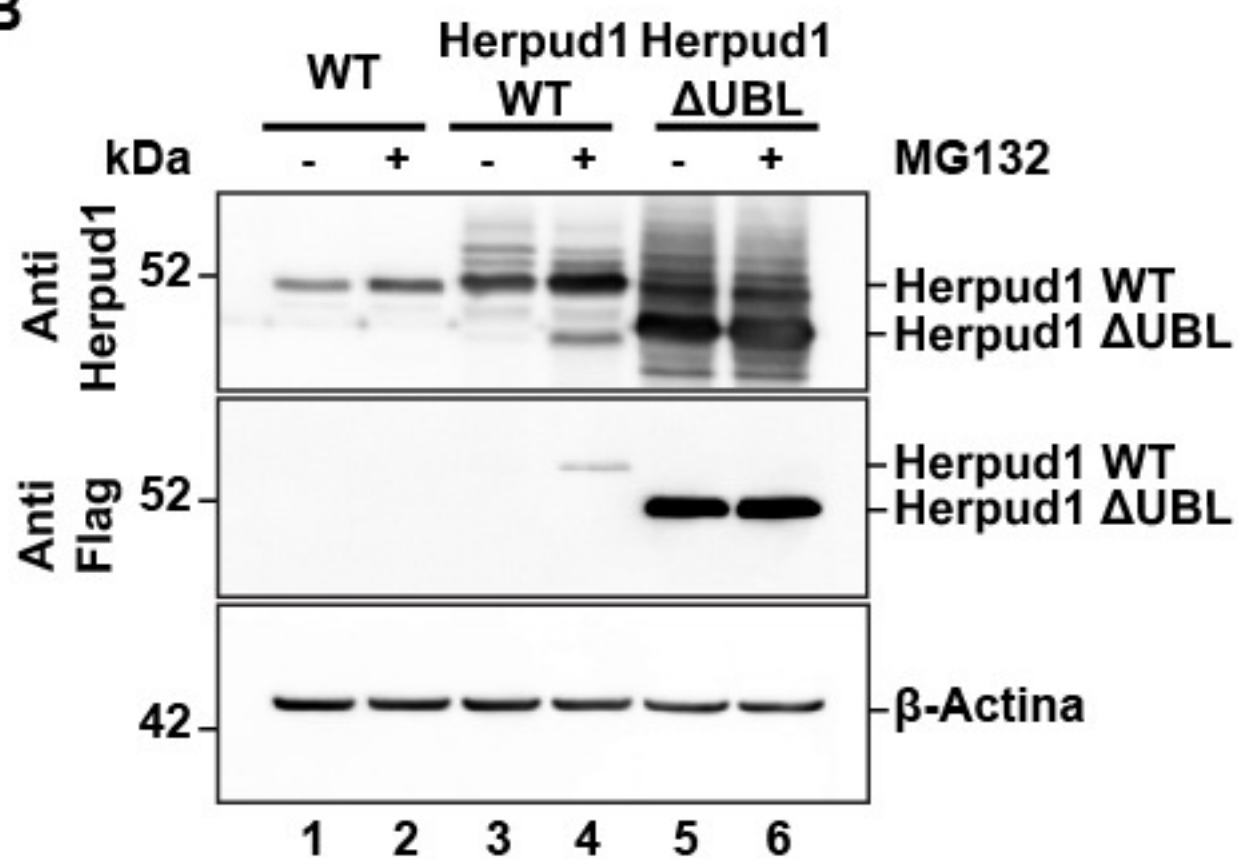

**A**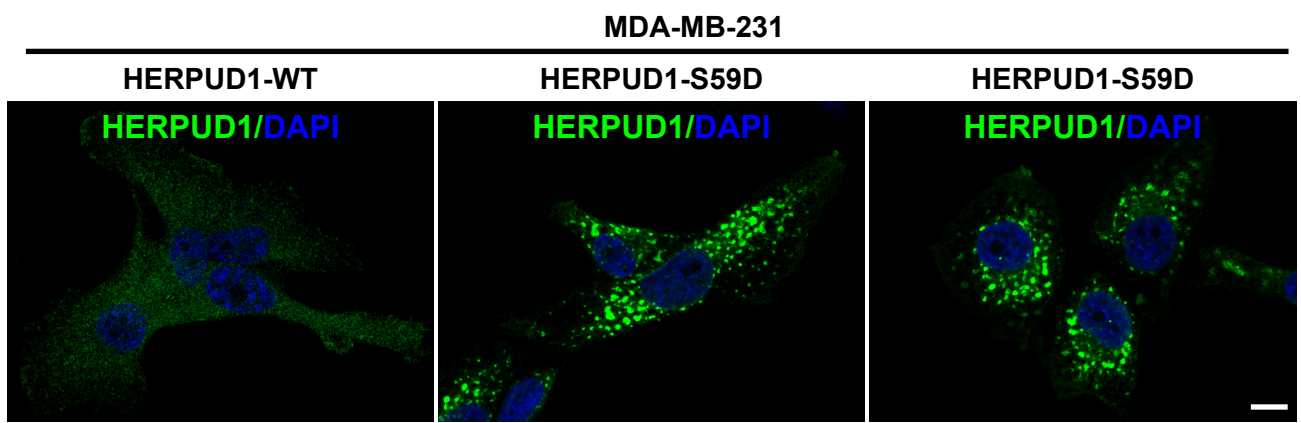**B**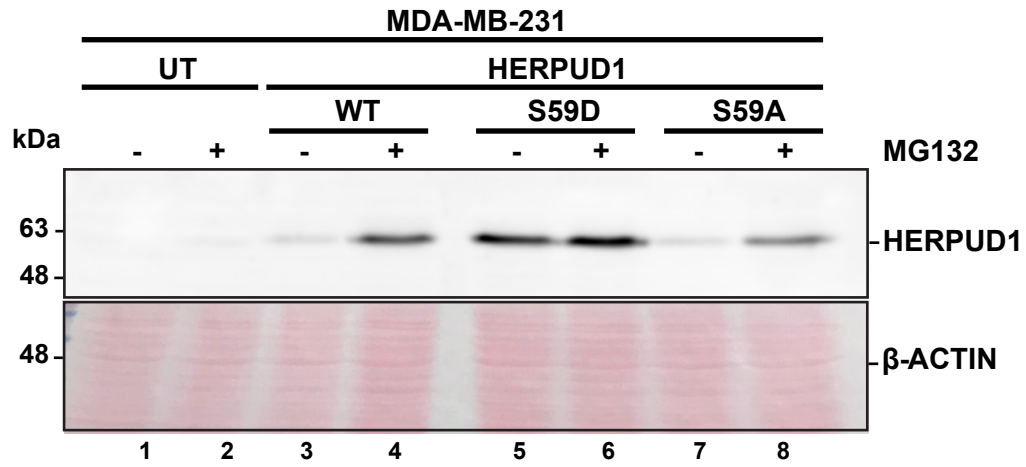

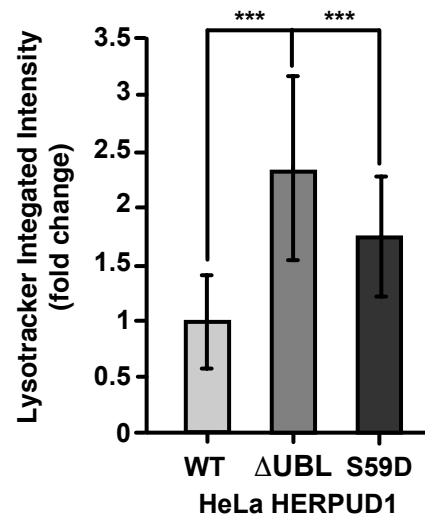

Supplement: Supplementary file 1 [file DataSheet4.PDF]

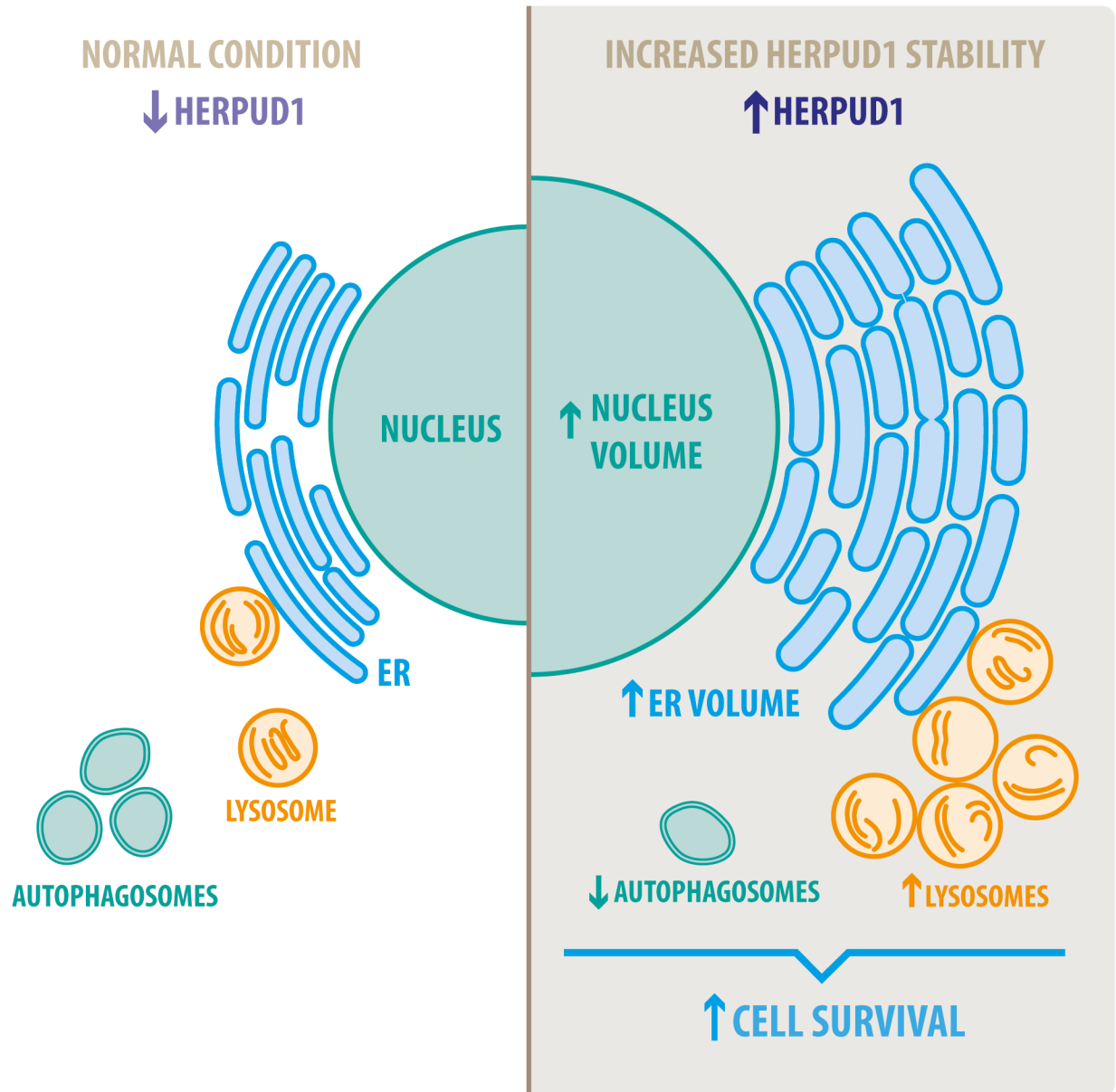

Supplement: Supplementary file 3 [file DataSheet1.PDF]
