# Supplementary material for: Negative Modulation of Macroautophagy by Stabilized HERPUD1 is Counteracted by an Increased ER-Lysosomal Network With Impact in Drug-Induced Stress Cell Survival
Source: Front Cell Dev Biol. 2022 Mar 2;10:743287. doi: 10.3389/fcell.2022.743287 (PMC8924303; doi:10.3389/fcell.2022.743287)
Supplement: Supplementary file 2 [file DataSheet3.PDF]

Gene names	Log2 Starvation /Norm shLuc	Log2 Starvation /Norm shATG5	Protein names
HERPUD1	-2.3755	-2.9502	Homocysteine-responsive endoplasmic reticulum-resident ubiquitin-like domain member 1 protein
SQSTM1	-1.9500	-1.7523	Sequestosome-1
RRM2	-1.7683	-1.4810	Ribonucleoside-diphosphate reductase subunit M2
CHCHD2;CHCHD2P9	-1.5471	-1.2523	Coiled-coil-helix-coiled-coil-helix domain-containing protein 2, mitochondrial
CYP1B1	-1.5041	-1.1918	Cytochrome P450 1B1
JAK1	-1.3608	-1.2387	Tyrosine-protein kinase JAK1
PIK3C3	-1.2089		Phosphatidylinositol 3-kinase
FAM127A		-1.1671	Protein FAM127A
UBE2S		-1.1280	Ubiquitin-conjugating enzyme E2 S
DSP	-1.1525	-1.0391	Desmoplakin
HMOX1	-1.3142	-0.7418	Heme oxygenase 1
UCK2	-1.1414	-0.9024	Uridine-cytidine kinase 2
DIDO1		-1.0206	Death-inducer obliterator 1
CCNB1		-0.9982	G2/mitotic-specific cyclin-B1
DNAJA1	-1.1604	-0.8325	DnaJ homolog subfamily A member 1
CTNNA2	-0.9902		Catenin alpha-2
PRSS56	-0.7467	-1.2140	Serine protease 56
DDX5	-1.0243	-0.8596	Probable ATP-dependent RNA helicase DDX5
CDKN2A	-1.0032	-0.8376	Cyclin-dependent kinase inhibitor 2A, isoforms 1/2/3
DNAJB4	-1.0796	-0.6709	DnaJ homolog subfamily B member 4
HSPA1A	-1.0232	-0.6260	Heat shock 70 kDa protein 1A/1B
HNRNPK	-0.7659	-0.8384	Heterogeneous nuclear ribonucleoprotein K
MAT2A	-0.8386	-0.7585	S-adenosylmethionine synthase isoform type-2
NTPCR	-1.3866	-0.2104	Cancer-related nucleoside-triphosphatase
CTNNA1	-0.7467	-0.7750	Catenin alpha-1
YTHDF2	-0.8372	-0.6683	YTH domain-containing family protein 2
LARP4	-1.0016	-0.4818	La-related protein 4
DFFA	-0.8074	-0.6549	DNA fragmentation factor subunit alpha
KPNA2	-0.8162	-0.6413	Importin subunit alpha-1
CSDE1	-0.7522	-0.6710	Cold shock domain-containing protein E1
PDLIM5	-0.7219	-0.6991	PDZ and LIM domain protein 5
SLC39A14	-0.7333	-0.6765	Zinc transporter ZIP14
EPPK1	-0.8574	-0.5506	Epiplakin
KPNA1	-0.5970	-0.8046	Importin subunit alpha-5
DNAJB1	-0.8146	-0.5555	DnaJ homolog subfamily B member 1
BAG3	-0.7414	-0.6209	BAG family molecular chaperone regulator 3
EHD4	-0.5058	-0.8238	EH domain-containing protein 4
VPS13A	-0.6579		Vacuolar protein sorting-associated protein 13A
CTTN	-0.7476	-0.5014	Src substrate cortactin
CKAP5	-0.7423	-0.4996	Cytoskeleton-associated protein 5
INF2	-0.7301	-0.5117	Inverted formin-2
DIABLO	-0.7455	-0.4922	Diablo homolog, mitochondrial
ZC3HAV1	-0.6140	-0.5688	Zinc finger CCCH-type antiviral protein 1
ASNS	-0.5693	-0.6099	Asparagine synthetase [glutamine-hydrolyzing]
TSR1	-0.7308	-0.4485	Pre-rRNA-processing protein TSR1 homolog
CYR61	-0.1370	-1.0238	Protein CYR61
PAK2	-0.5284	-0.6034	Serine/threonine-protein kinase PAK 2
FXR1	-0.5265	-0.6019	Fragile X mental retardation syndrome-related protein 1
EIF5	-0.4853	-0.6181	Eukaryotic translation initiation factor 5
SSRP1	-0.7011	-0.3745	FACT complex subunit SSRP1
PICALM	-0.3923	-0.6463	Phosphatidylinositol-binding clathrin assembly protein
RRM1	-0.7963	-0.1904	Ribonucleoside-diphosphate reductase large subunit
KRT18	-0.6322	-0.3419	Keratin, type I cytoskeletal 18
TPT1	-0.6253	-0.3430	Translationally-controlled tumor protein
EIF4B	-0.4945	-0.4216	Eukaryotic translation initiation factor 4B
DDX3X;DDX3Y	-0.4678	-0.3529	ATP-dependent RNA helicase DDX3X
DDX17	-0.4658	-0.3408	Probable ATP-dependent RNA helicase DDX17
WARS	-0.4621	-0.3410	Tryptophan--tRNA ligase, cytoplasmic
HSPA6	-0.4815	-0.3151	Heat shock 70 kDa protein 6
LMNB1	-0.4677	-0.2722	Lamin-B1
HSPH1	-0.4016	-0.2609	Heat shock protein 105 kDa
NONO	-0.3935	-0.2097	Non-POU domain-containing octamer-binding protein
CENPF	-0.5236	0.0339	Centromere protein F
KPRP	0.7616	-1.1857	Keratinocyte proline-rich protein
S100A8	0.3830	-0.5917	Protein S100-A8
PDLIM7	-0.4601	0.6020	PDZ and LIM domain protein 7
APOBEC3C	-0.6987	1.1531	DNA dC->dU-editing enzyme APOBEC-3C
HNRNPA2B1	0.4558	0.1988	Heterogeneous nuclear ribonucleoproteins A2/B1
SYPL1	0.9425	-0.2530	Synaptophysin-like protein 1
NOP56	0.8012	-0.0723	Nucleolar protein 56
EFTUD2	0.4682	0.3682	116 kDa U5 small nuclear ribonucleoprotein component
TCOF1	0.5133	0.3271	Treacle protein
SNRPA1	0.4857	0.4099	U2 small nuclear ribonucleoprotein A
BCCIP	-0.0579	0.9899	BRCA2 and CDKN1A-interacting protein
DDAH2	0.8945	0.0728	N(G),N(G)-dimethylarginine dimethylaminohydrolase 2
SLC4A7	0.2193	0.7783	Sodium bicarbonate cotransporter 3
PSIP1	0.7405	0.3509	PC4 and SFRS1-interacting protein
HIST1H1E	0.7938	0.3070	Histone H1.4
BRIX1	0.7510	0.4148	Ribosome biogenesis protein BRX1 homolog
HIST1H1C;HIST1H1D	0.7096	0.4598	Histone H1.2
FAM96A	-0.0920	1.3367	MIP18 family protein FAM96A
MRTO4	0.7314	0.5427	mRNA turnover protein 4 homolog
GTPBP4	1.0446	0.2341	Nucleolar GTP-binding protein 1
HIST1H1B	0.8032	0.5083	Histone H1.5
CXADR	1.4132	-0.0661	Coxsackievirus and adenovirus receptor
MAN2A1	0.2853	1.1192	Alpha-mannosidase 2
GYG1	0.8479	0.5741	Glycogenin-1
HMGN1	1.0140	0.4165	Non-histone chromosomal protein HMG-14
GLI2	0.6774	0.7593	Zinc finger protein GLI2
PKP3	-0.5195	1.9642	Plakophilin-3
DSG1		0.7253	Desmoglein-1
PDCD4	0.6855	0.8296	Programmed cell death protein 4
NOP2	1.0216	0.5255	Probable 28S rRNA (cytosine(4447)-C(5))-methyltransferase
ALDH1A3	1.4997	0.0761	Aldehyde dehydrogenase family 1 member A3
TAF15	0.8080		TATA-binding protein-associated factor 2N
MRPS26	1.4780	0.1647	28S ribosomal protein S26, mitochondrial
ERC1	0.8614		ELKS/Rab6-interacting/CAST family member 1
HMGN3;HMGN2	0.9721	0.7621	High mobility group nucleosome-binding domain-containing prot. 3
DDX27	1.0440	0.7351	Probable ATP-dependent RNA helicase DDX27
VIM	0.7521	1.1510	Vimentin
RSL1D1	1.2941	0.7582	Ribosomal L1 domain-containing protein 1
RALY	1.2345	0.8586	RNA-binding protein Raly
FBL	1.6435	0.8101	rRNA 2-O-methyltransferase fibrillarin
HNRNPC;HNRNPCL1	1.5508	0.9595	Heterogeneous nuclear ribonucleoproteins C1/C2
NUMA1	1.4976	1.0158	Nuclear mitotic apparatus protein 1
SBSN	1.3332		Suprabasin
H2AFV;H2AFZ	1.7766	1.3893	Histone H2A.V
H3F3A	2.0758	1.8649	Histone H3.3
HIST1H2AC;HIST3H2A;HIST1H2AB	2.5651	2.0782	Histone H2A type 1-C
HIST2H3A	2.8848	1.8520	Histone H3.2
HIST1H2BL;HIST1H2BM;HIST1H2BN;HIST1H2BH;HIST2H2BF;HIST1H2BC;HIST1H2BD;HIST1H2BK;H2BFS	2.9014	2.2069	Histone H2B
HIST1H4A	2.8226	2.3639	Histone H4
HIST2H2AC;HIST2H2AA3	2.9450	2.2908	Histone H2A type 2-C
HIST2H2BE;HIST1H2BB;HIST1H2BO;HIST1H2BJ;HIST3H2BB	3.0875	2.3765	Histone H2B type 2-E
HIST2H3PS2	2.8909	3.0398	Histone H3
H2AFY	3.1737	2.8956	Core histone macro-H2A.1
HIST1H3A;HIST3H3;H3F3A;H3F3C	3.3200	2.7767	Histone H3.1

Supplementary Figure 1

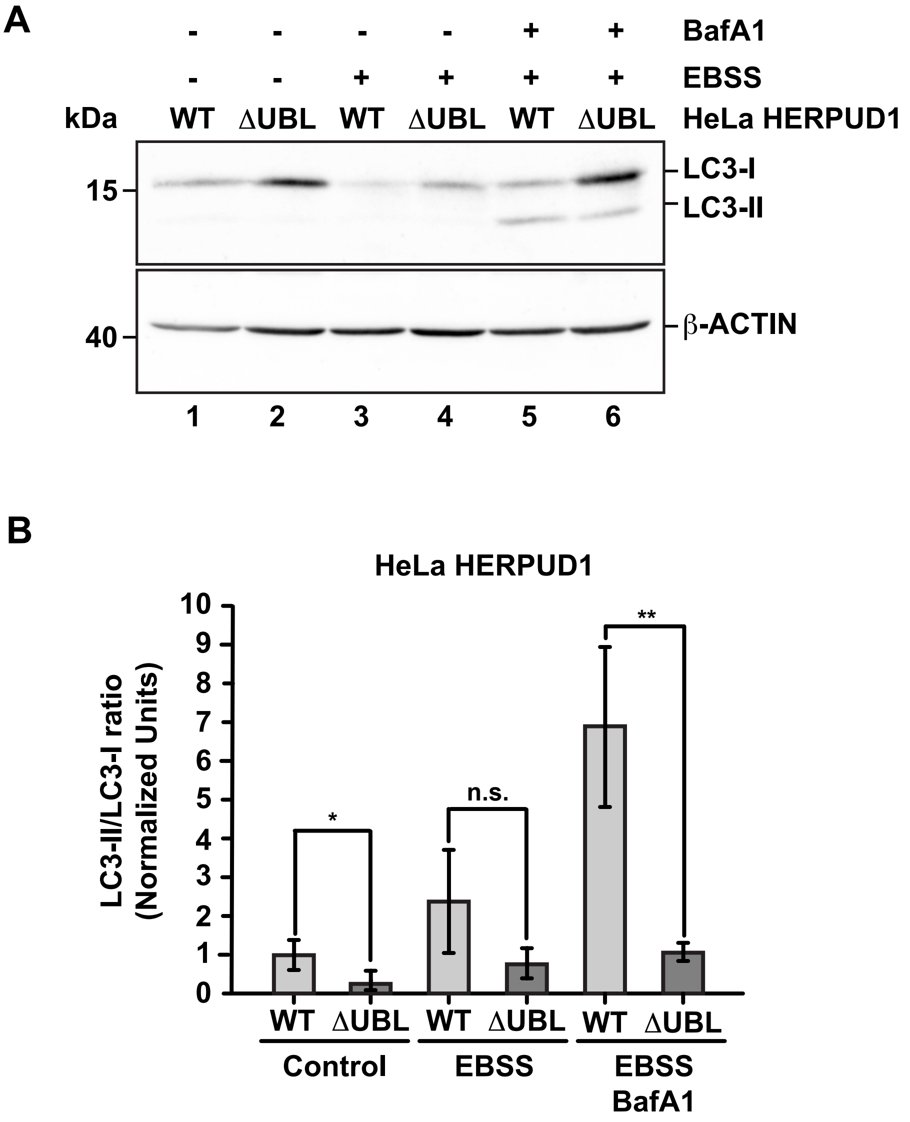

Supplementary Figure 2

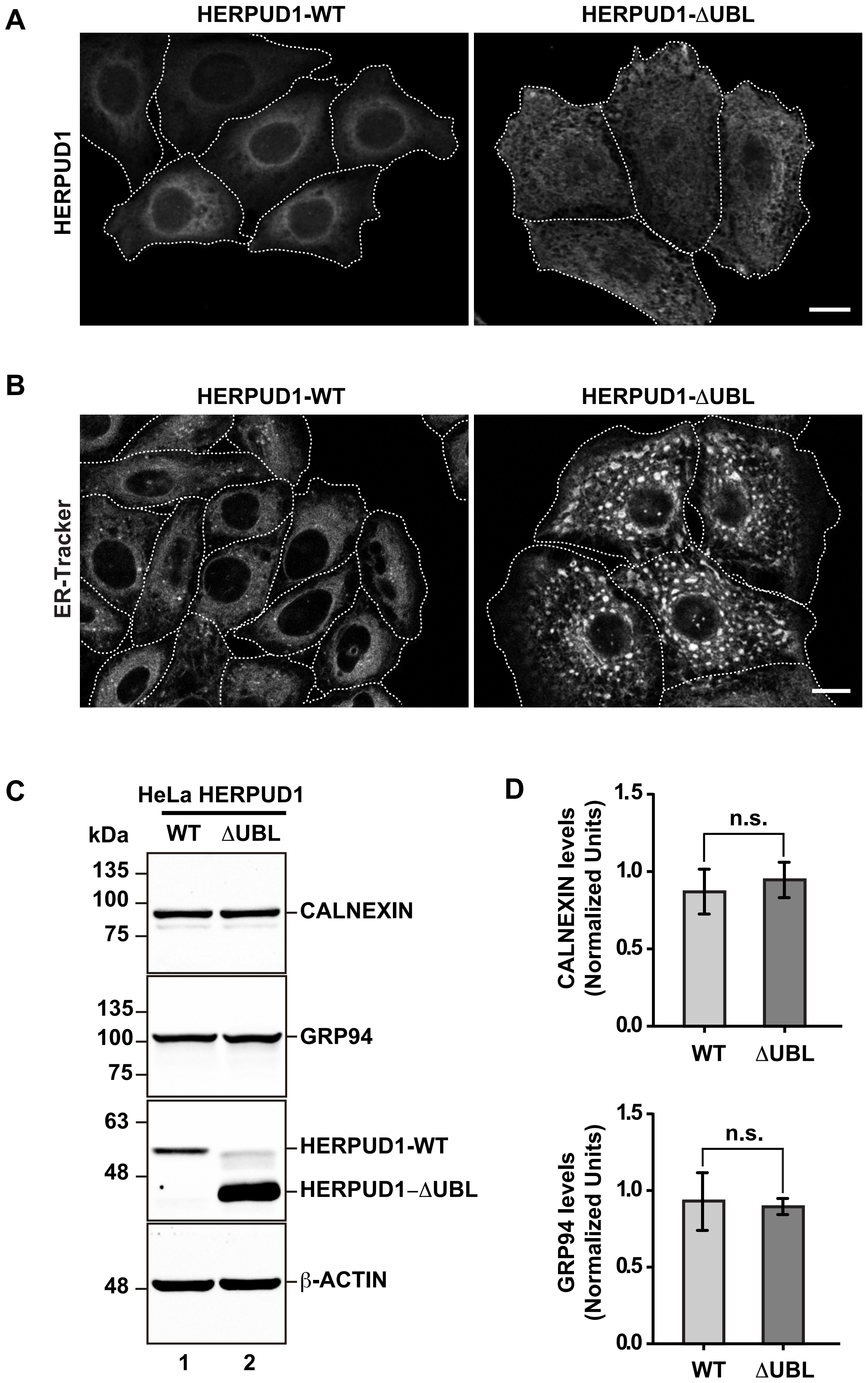

Supplementary Figure 3

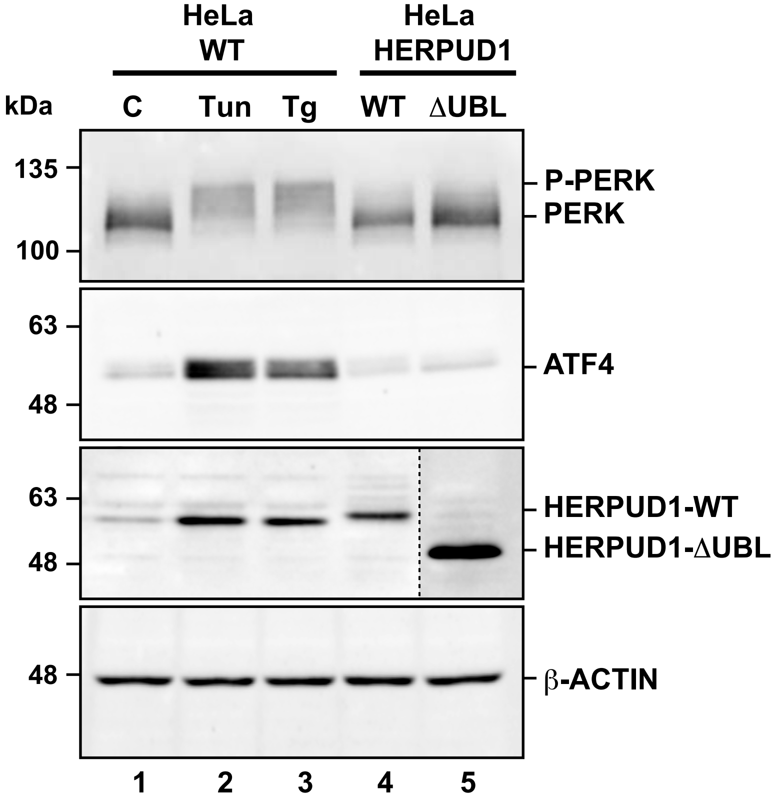

Supplementary Figure 4

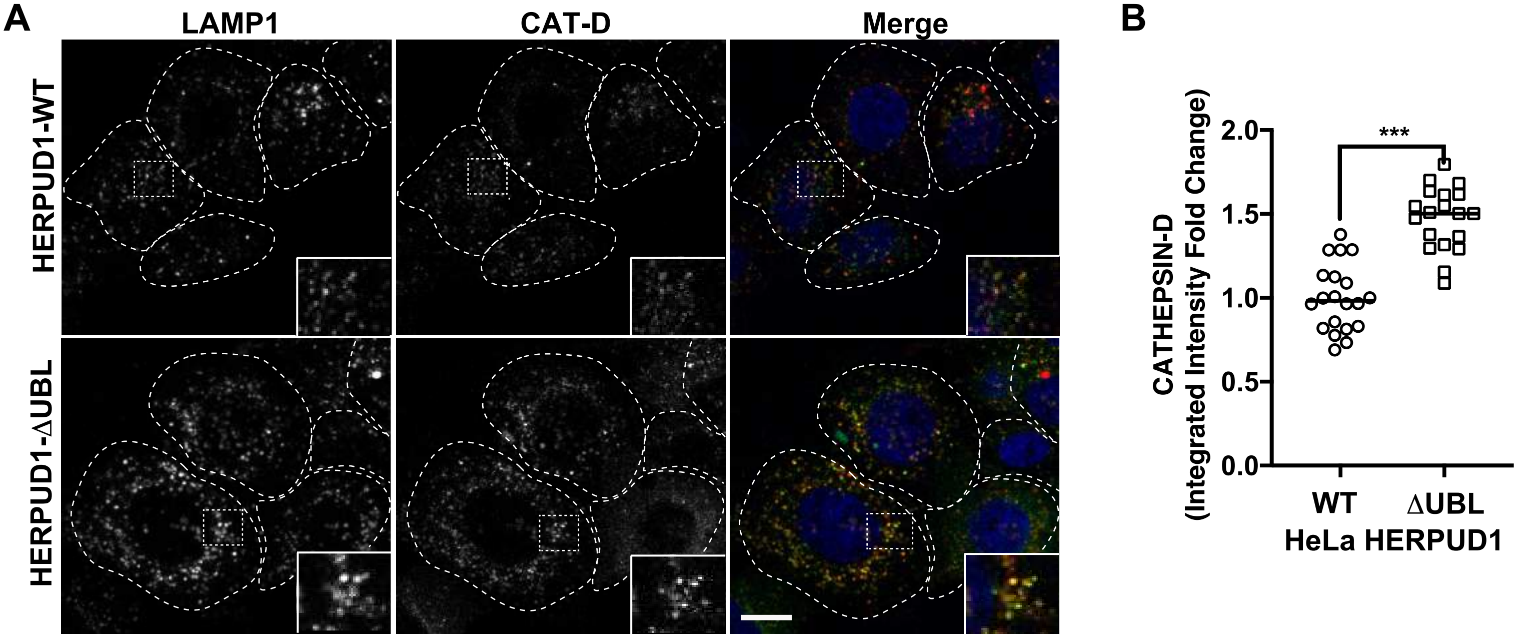

Supplementary Figure 5

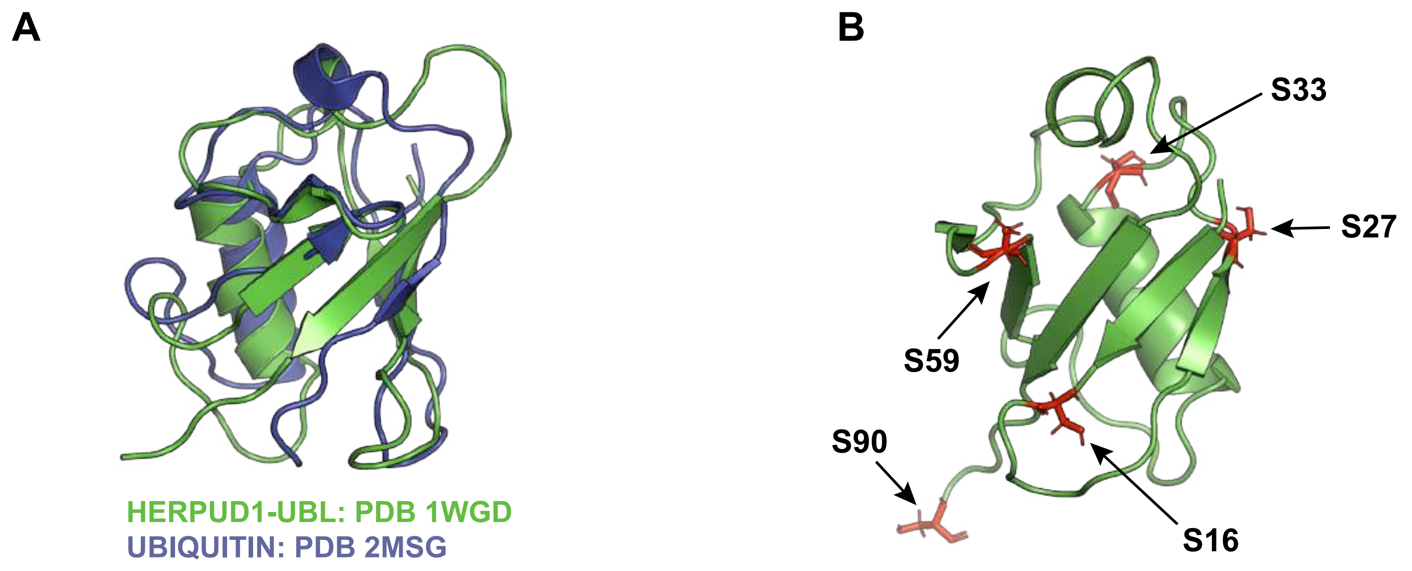

Supplementary Figure 6

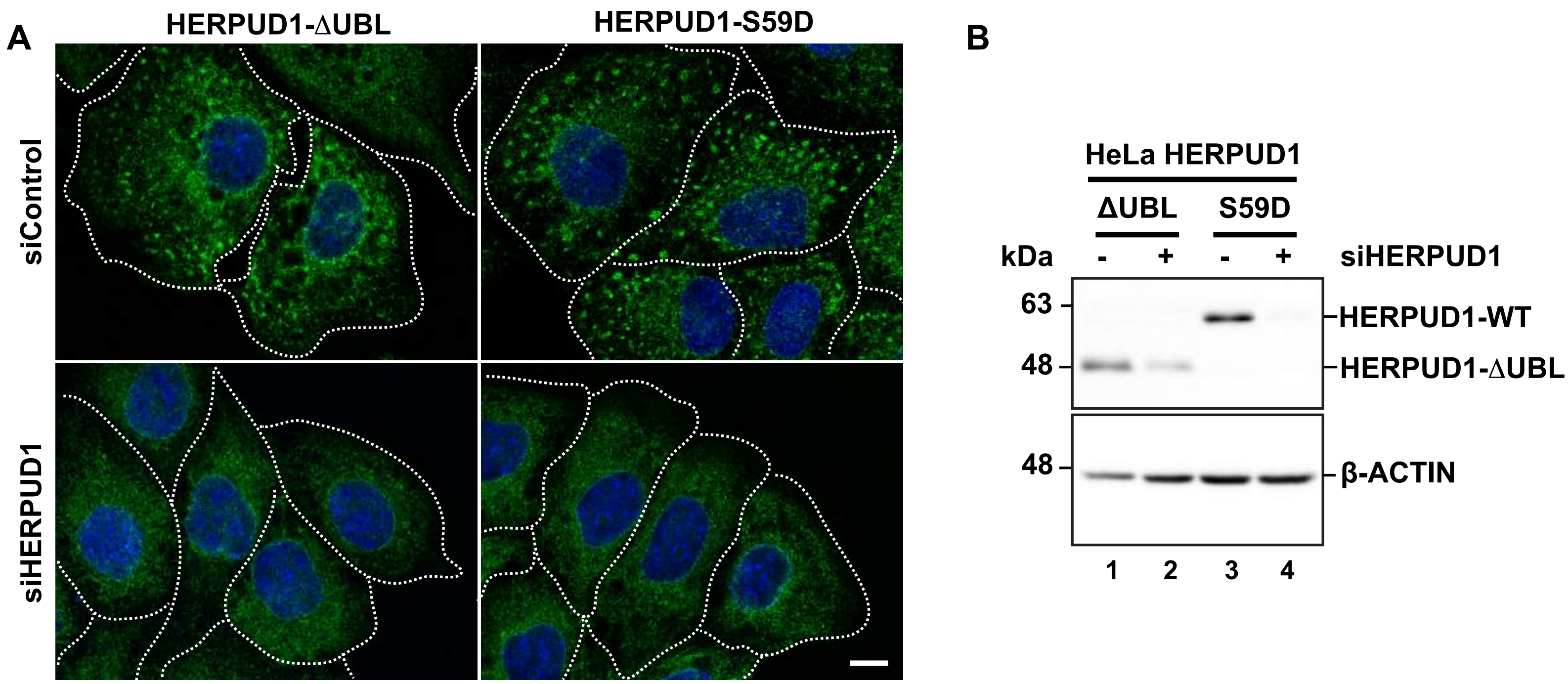

Supplementary Figure 7

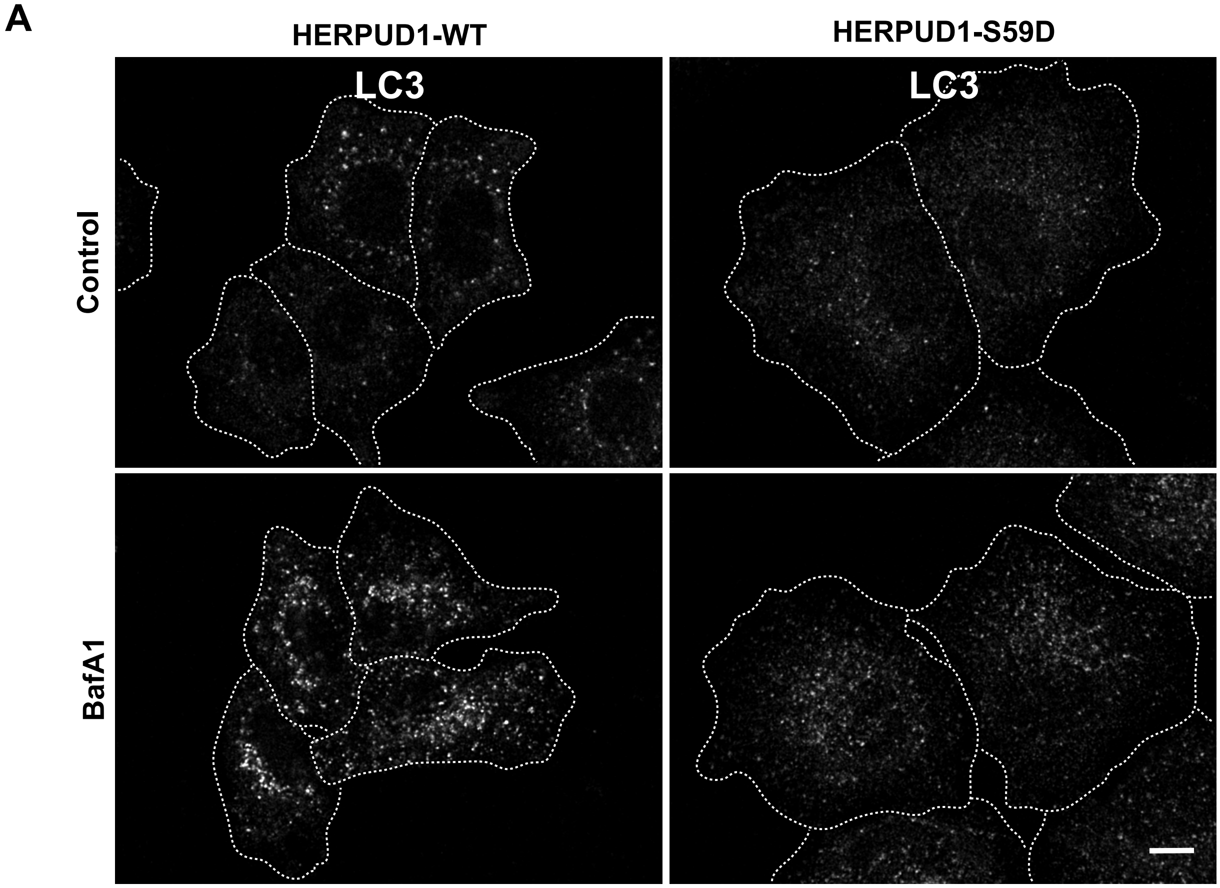

Supplementary Figure 8

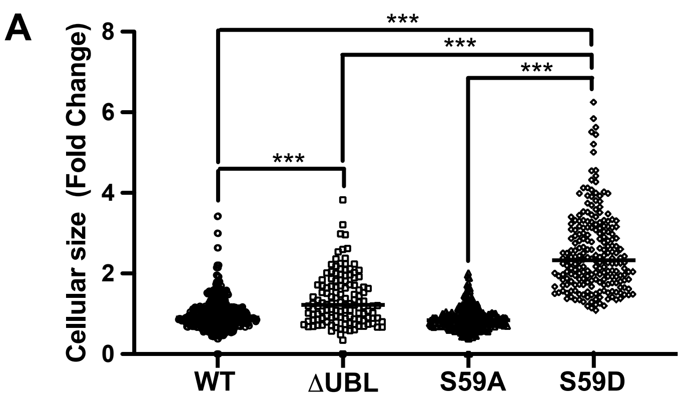

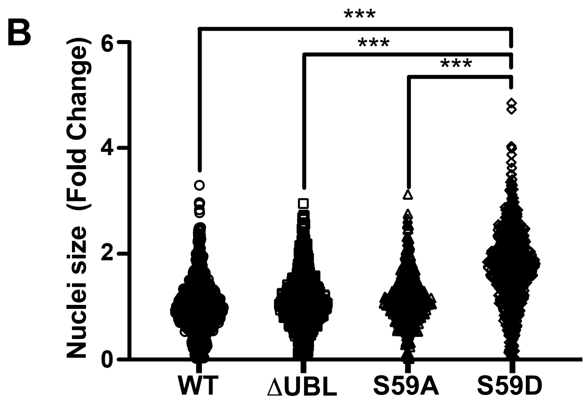

Supplementary Figure 9

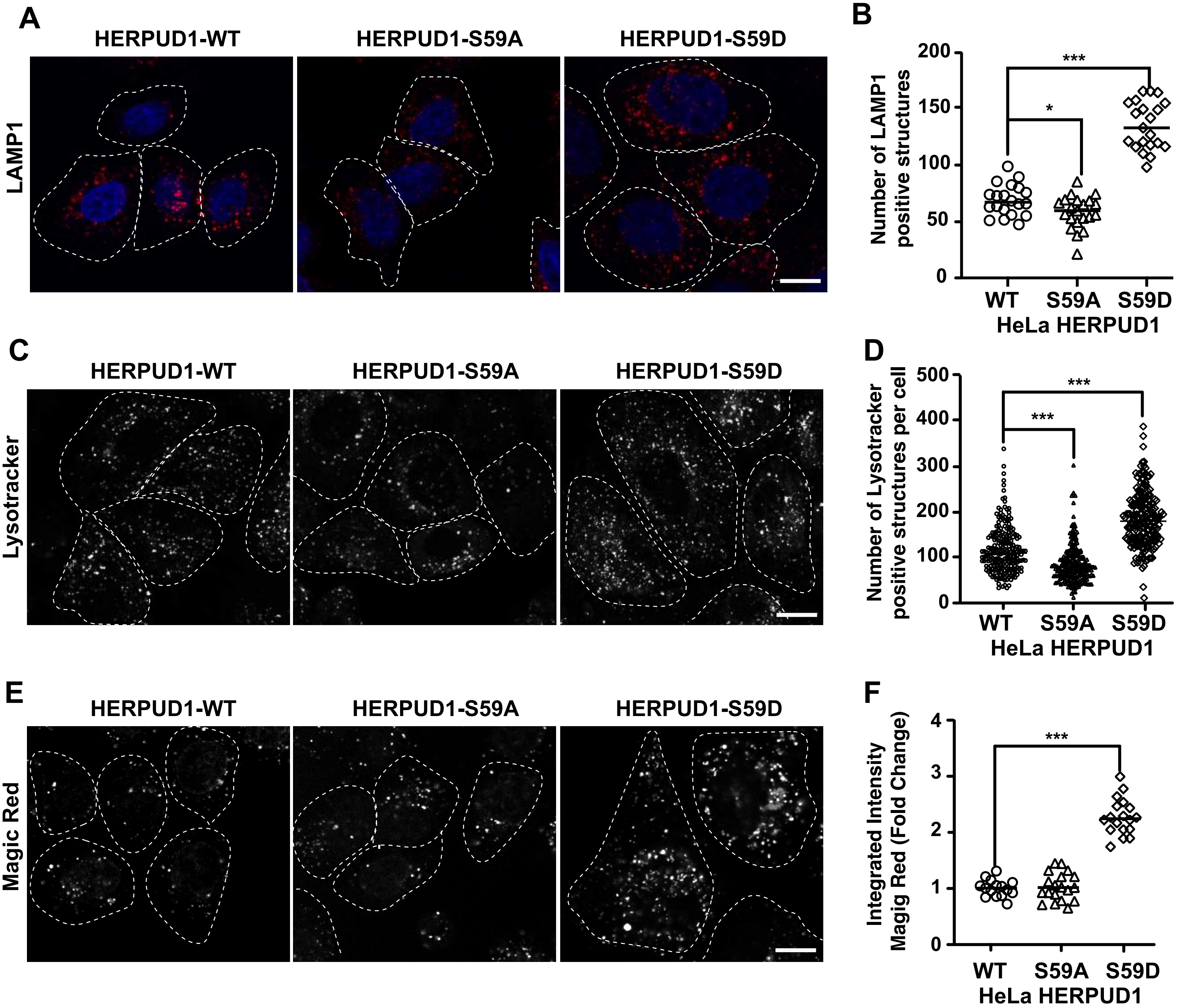

Supplementary Figure 10
